# Bundle care approach to reduce device associated infections in post-living-donor-liver transplantation in a tertiary care hospital, Egypt

**DOI:** 10.1186/s12879-024-09525-4

**Published:** 2024-07-05

**Authors:** Mona A. Wassef, Doaa M. Ghaith, Marwa M. Hussien, Mostafa A. El-Shazly, Reham H. A. Yousef

**Affiliations:** 1 Clinical and Chemical Pathology Department, Faculty of Medicine, Cairo University, Al-Saray Street, Al-Manial, Cairo, 11559 Egypt; 2 General surgery and liver transplantation, Faculty of Medicine, Cairo University, Cairo, Egypt; 3 Microbiology, Immunology and Infectious Disease Department, College of Medicine and Medical Sciences, Arabian Gulf University, Manama, Bahrain

**Keywords:** Liver transplantation, Infections associated with devices, Bundle care, Central lines, CAUTI, VAP

## Abstract

**Background:**

Device-associated infections (DAIs) are a significant cause of morbidity following living donor liver transplantation (LDLT). We aimed to assess the impact of bundled care on reducing rates of device-associated infections.

**Methods:**

We performed a before-and-after comparative study at a liver transplantation facility over a three-year period, spanning from January 2016 to December 2018. The study included a total of 57 patients who underwent LDLT. We investigated the implementation of a care bundle, which consists of multiple evidence-based procedures that are consistently performed as a unified unit. We divided our study into three phases and implemented a bundled care approach in the second phase. Rates of pneumonia related to ventilators [VAP], bloodstream infections associated with central line [CLABSI], and urinary tract infections associated with catheters [CAUTI] were assessed throughout the study period. Bacterial identification and antibiotic susceptibility testing were performed using the automated Vitek-2 system. The comparison between different phases was assessed using the chi-square test or the Fisher exact test for qualitative values and the Kruskal-Wallis H test for quantitative values with non-normal distribution.

**Results:**

In the baseline phase, the VAP rates were 73.5, the CAUTI rates were 47.2, and the CLABSI rates were 7.4 per one thousand device days (PDD). During the bundle care phase, the rates decreased to 33.3, 18.18, and 4.78. In the follow-up phase, the rates further decreased to 35.7%, 16.8%, and 2.7% PDD. The prevalence of *Klebsiella pneumonia* (37.5%) and Methicillin resistance *Staph aureus* (37.5%) in VAP were noted. The primary causative agent of CAUTI was *Candida albicans*, accounting for 33.3% of cases, whereas Coagulase-negative *Staph* was the predominant organism responsible for CLABSI, with a prevalence of 40%.

**Conclusion:**

This study demonstrates the effectiveness of utilizing the care bundle approach to reduce DAI in LDLT, especially in low socioeconomic countries with limited resources. By implementing a comprehensive set of evidence-based interventions, healthcare systems can effectively reduce the burden of DAI, enhance infection prevention strategies and improve patient outcomes in resource-constrained settings.

## Introduction

Patients afflicted with hepatocellular carcinoma, chronic failure, or end-stage liver disease have liver transplantation (LT) as their efficacious last-line therapy [1]. The prevalence of hepatitis C virus (HCV) has led to an increase in the number of patients with chronic liver disease in Egypt [2]. 

The prognostic implications of DAIs in LT are significant. Studies have shown that LT recipients who develop DAIs have a higher risk of mortality, as these infections can lead to sepsis and multi-organ dysfunction [3]. Infections can also adversely affect the long-term survival and function of the transplanted liver, increasing the risk of graft failure and the need for re-transplantation [4]. Additionally, the management of DAIs is associated with prolonged hospital stays, intensive care utilization, and substantial economic burden on healthcare systems, especially in resource-limited settings. Furthermore, recurrent or persistent DAIs can contribute to the development and spread of antimicrobial-resistant pathogens, posing a broader public health concern [5]. DAIs constitute a significant cause of morbidity following LDLT, with bacterial infections accounting for approximately 70%, followed by viral and fungal infections at 20 and 8%, respectively [6]. Understanding these prognostic implications is crucial for healthcare providers to prioritize infection prevention and optimize management strategies to improve patient outcomes in LT.

Following the Centre for Disease Control and Prevention ( CDC’s) confirmation that nearly 35% of hospital-acquired infections (HAIs) can be prevented through the implementation of effective infection prevention and control programs, numerous researchers have investigated various methods of surveillance programs to reduce HAIs [7].

Consistently implementing the care bundle method enhances patient outcomes. The care bundle method has been effectively implemented in the surgical intensive care units (ICU). Nevertheless, the extent to which it effectively decreases the incidence of HAIs in LDLT remains undetermined [7, 8].

Our study aimed to evaluate the impact of implementing bundled care on reducing DAI rates. Furthermore, we aimed to identify bacterial infection and antibiotic resistance patterns in LDLT at Almanial Specialized University Teaching Hospital, Cairo University.

## Materials and methods

### Study setting and design

This study is a retrospective comparative study conducted from January 2016 to December 2018 at the liver transplantation unit, Almanial Specialized University Teaching Hospital, Cairo University. The hospital includes 328 beds distributed over different sections and eight ICUs. The liver transplantation Unit has four isolation rooms and one ICU. There are approximately 20–24 patients who undergo LDLT operations annually. The study included 57 patients admitted to LT and underwent LDLT operations.

We investigated the effect of implementing multiple evidence-based procedures grouped as a single care bundle and performed constantly as one unit. We divided the study into three different phases.

### Phase 1: baseline assessment

This phase was conducted over 15 months. A total of 24 patients underwent LDLT during this period. The infection rates associated with central lines, urinary catheters, and ventilators (CLABSI, CAUTI, VAP) were evaluated in all patients using the standard surveillance method for HAI as defined by the CDC 2017 [9].

### Phase 2: implementation of care bundles

The duration of this phase spanned six months and encompassed a cohort of 12 patients who underwent LDLT. The care bundles were designed in accordance with the CDC guidelines [8, 10] and presented to the infection control team of the Liver transplantation ICU. The healthcare workers in the infection control team received health education, which varies from one-to-one and on-the-job training. This education was conducted by a single experienced researcher on a daily basis during designated working hours. The researcher delivered a 20-minute mass lecture using CDC materials. It occurred daily at the beginning of every shift to all the nurses on duty. It included the component elements of the bundle and emphasized the significance of consistently employing them in conjunction.

The component elements of the care bundle for preventing catheter-associated UTI are as follows: maximal personal protective equipment during catheter insertion, sterile closed drainage system, the urinary catheter is never disconnected, the collecting bag below bladder level, evacuating the urinary collecting bag every hour to maintain less than 75% full. The component elements of the care bundle to prevent central line BSI are as follows: adherence to HH compliance before any manipulations with the catheter, using maximum sterile personal protective equipment, using chlorhexidine/betadine skin antisepsis, daily inspection for local signs of infections by the good condition of sterile dressing, daily checking of the necessity for catheter presence, and using flushing only once. The component elements of the care bundle to prevent VAP included the following: 30–50 degree raising of the bed head, daily checking of the ability to wean, suctioning of the subglottic area three times per day, ensuring endotracheal cuff pressure a minimum of 20 cm, oral care with an antiseptic solution, prophylaxis for stress ulcers and deep vein thrombosis.

The hospital infection control team calculated the rates of DAIs in all patients who underwent LDLT using the following formula: Total DAI incidence in LDLT = No. of infections/total No. of device days × 1000. We monitored the compliance of the healthcare workers’ adherence to all the components of the care bundle together.

### Phase 3: follow-up assessment

This phase spanned 15 months and included 21 patients who underwent LDLT. It occurred immediately following the phase of care bundle implementation. The use of bundled care forms was continued. The health education activities were reiterated. A systematic monitoring of DAI rates was carried out to determine whether infection rates showed any improvement following the implementation. The researchers performed an antibiogram at various phases of the study.

Furthermore, we monitored the rate of adherence to hand hygiene protocols (HH) at the baseline, during the implementation of the care bundles, and during the follow-up period. Observations included the five moments suggested by the World Health Organisation (WHO): before and after patient contact, before any aseptic procedure, and subsequent to any contact with body fluids and patient surroundings.

### Microbiology work-up

Prior to surgery, all patients underwent preoperative screening to detect colonization of methicillin-resistant Staphylococcus aureus (MRSA) and Gram-negative bacteria producing extended-spectrum beta-lactamase (ESBL). The screening process involved collecting nasal, axillary, and groin swabs from each patient for MRSA whereas rectal swabs for ESBL detection. All collected swabs were inoculated onto chromogenic media (CHROMagar, France).

Urine, endotracheal aspirates, and blood cultures were cultured on MacConkey, chocolate, blood agar (Oxoid, England). After incubation for 24 h at 35 °C, all bacterial strains were identified and checked for antibiotic sensitivity using the Vitek2 compact (Biomerieux, France) according to the manufacturer’s guidelines. All clinical isolates underwent phenotypic confirmation, including approximation test using Cefotaxime and Amoxicillin-clavulanic acid discs for ESBL detection in Gram-negative bacteria, and a Cefoxitin sensitivity test for MRSA confirmation. The results were interpreted using the guidelines provided by the Clinical Laboratory Standards Institute (CLSI). The terms multidrug (MDR), pan-drug (PDR), and extreme drug-resistant (XDR) organisms were defined according to Magiorakos et al. and CLSI [11, 12].

### Statistical analysis

The Statistical Package for the Social Sciences (SPSS; IBM et al., USA) version 22 for Microsoft Windows was used for data analysis. Data were expressed as mean ± standard deviation, median, and range for quantitative variables. In addition, qualitative variables were expressed as frequency and percentages. The comparison between different phases was assessed using the chi-square test or the Fisher exact test for qualitative values and the Kruskal-Wallis H test for quantitative values with non-normal distribution. The Kruskal-Wallis test was utilized to compare age between the different groups, as the age data were non-normally distributed.

Within-group comparisons were conducted using the McNemar test. *P*-values < 0.05 were considered statistically significant.

### Ethical approval and consent to participate

Both The Ethical Committee of the Department and Cairo University approved the study. All patients gave their written informed consent after explaining the study’s aim and importance following the declaration of Helsinki. All patient data were treated with confidentiality throughout the phases of the study.

## Results

The mean ages of liver recipients in the first and follow-up phases were 49.75 and 34.62 years, respectively. The mean ages of the donors were 32.08 and 30.1 years in the same two phases, respectively. Hepatocellular carcinoma (HCC) represented LT’s most common predisposing factor across all phases, followed by hepatitis C virus (HCV)-related liver cirrhosis, Budd–Chiari syndrome, and autoimmune hepatitis. HCV was the most prevalent viral infection in preoperative viral screening of 75% of the patients in the baseline assessment and bundle implementation phases and 66% in the follow-up phase. No patients were found positive for Human Immunodeficiency Virus (HIV) and Epstein-Barr Virus (EBV) after screening across all study phases. In contrast, there were no significant effects of age, sex, or underlying diseases on the rates of infections in any of the study phases as shown in Table 1.

**Table 1:** Demographic data, comorbidity, viral screening, and underlying chronic diseases

	Base line *N* 24 (%)	Implementation *N* 12(%)	Follow up *N* 21(%)	Total *N* 57 (%)		*P* value
Male Female	19 (79.2) 5(20.8)	12 (100) 0	19 (90.5) 2(9.5)	50 7	87.7 12.3	0.017
Age *	44(12.5)	53(14.5)	41(48)	44(17)		0.060
HCC	13 (54.2)	5 (41.7)	14(66.7)	32	56.1	0.026
liver cirrhosis	5(20.8)	7(58.3)	1(4.5)	13	22.8	
Budd-Chiari syndrome	5(20.8)	0	4(19)	9	15.8	
Autoimmune hepatitis	1(4.2)	0	2(9.5)	3	5.3	
HCV	18(75)	9 (75)	11(52.4)	38	66.7	0.21
HBV	1(4.2)	1(8.3)	1(4.8)	3	5.3	
CMV	0	0	1(4.8)	1	1.7	
DM	6(25)	1(8.3)	3(14.2)	10	17.5	0.60
HTN	5(20.8)	3(25)	1(4.7)	9	15.7	0.25

*Age is expressed as median (IQR) and compared using Kruskal Wallis Test, HCC: hepatocellular carcinoma; HCV: Hepatitis C virus; HBV: Hepatitis B Virus; CMV: cytomegalovirus,; DM: Diabetes Mellitus; HTN: hypertension

Our study showed that the consistent application of all the care bundle elements resulted in a reduction of more than 50% of the VAP, 64% of the CAUTI, and 63% of the CLABSI. When comparing the baseline assessment to the follow-up phase, the *p*-values were 0.45 for VAP, 0.28 for CAUTI, and 0.62 for CLABSI. Reductions in DAIs following care bundle implementation are demonstrated in Table 2 .

**Table 2:** VAP, CAUTI, CLABSI rates before and after the care bundle

phase		VAP rate			CAUTI rate			CLABSI rate		
	No. of patients	No. of VAP	ventilator days	VAP rate	No. of CAUTI	Catheter days	CAUTI rate	No. of CLABSI	Central line days	CLABSI rate
Base line	24	5	68	73.5	6	127	47.2	3	405	7.4
Implement.	12	1	30	33.3	1	55	18.18	1	209	4.78
Follow up	21	2	56	35.7	2	119	16.8	1	360	2.77
Total	57	8	154	-	9	301	-	5	974	-

VAP rate = VAP events/total number of ventilator days × 1000, CLABSI rate = CLABSI events/total number of central line days × 1000, CAUTI rate = CAUTI events/ total number of urinary catheter days × 1000, VAP = Ventilator-associated pneumonia, CLABSI = Central line-associated bloodstream infection, CAUTI = Catheter-associated urinary tract infection

The application of our ongoing health education program during all three stages of the study resulted in a significant adherence to hand hygiene, increasing from 57.3% at baseline to 78%. The median rate remained constant during the last six months of the follow-up period at 78%.

**Fig. 1:**
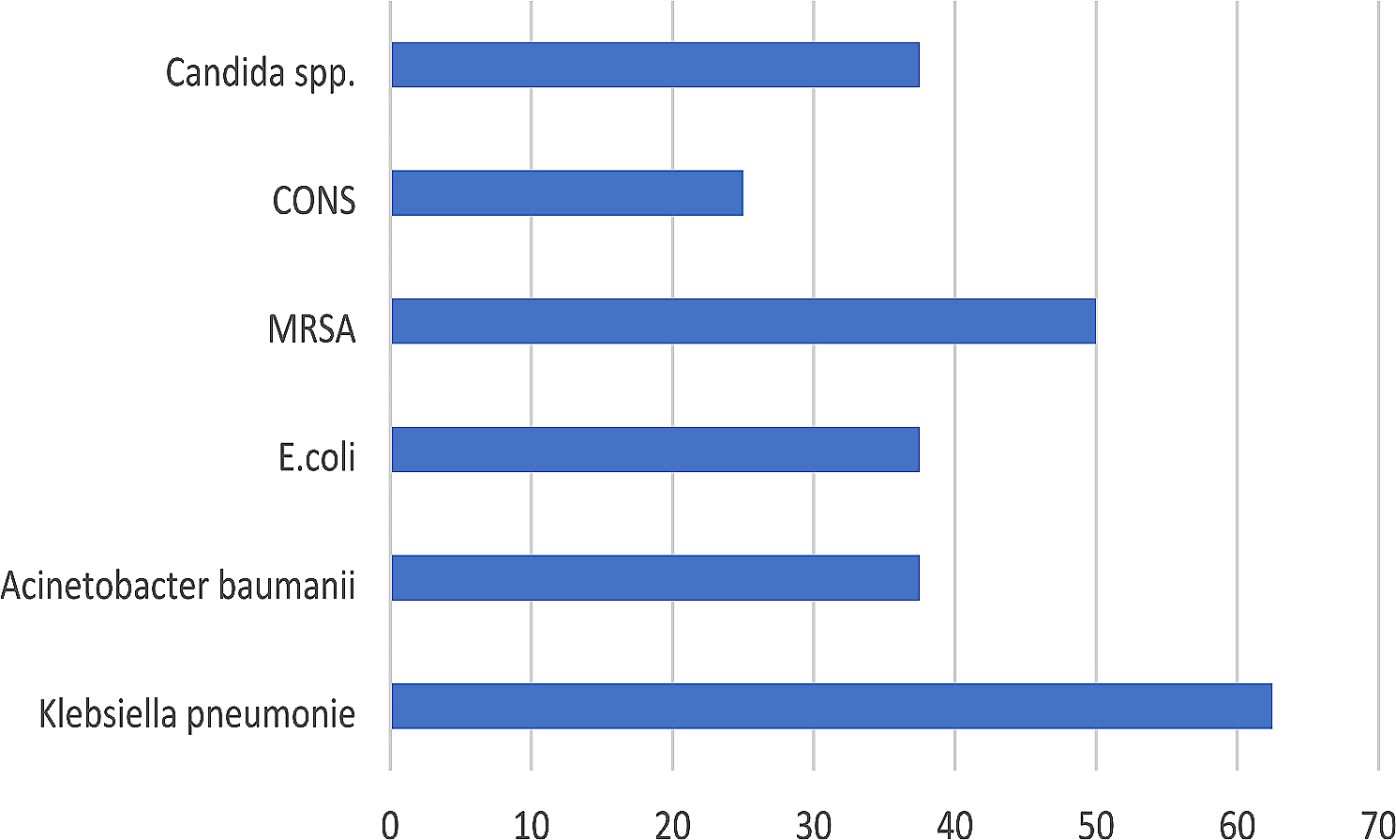
The distribution of pathogens of DAI in the liver transplantation intensive -care unit (numbers are presented as percentages)

In our study, we found nine cases of CAUTI, eight VAP events, and five CLABSI events, with different resistance patterns detected among the isolated organisms. The predominant resistance patterns were XDR, ESBL, and MRSA. CAUTI represented the most common DAI in patients with LDLT. The causative organisms for CAUTI were *Candida albicans* in 4/9 (44.4%) events, *Klebsiella pneumonia* in 2/9 (22.2%), *E. coli* in 2/9 (22.2%), and *Acinetobacter baumannii* in 1/9 (11%). VAP represented the second common DAI in patients with LDLT. *Klebsiella pneumonia* was responsible for 37.5% of the events in VAP, while Methicillin resistance *Staph aureus* (*MRSA)* was detected in 3/8 (37.5%), *Acinetobacter baumannii* in 1/8 (12.5%), and *Escherichia coli* in 1/8 (12.5%). The organisms that caused CLABSI were Coagulase-negative *Staph* in 2/5 (40%) events, *Klebsiella pneumonia* in 1/5 (20%), *Acinetobacter baumannii* in 1/5 (20%), and MRSA in 1/5 (20%). Collectively In our study, Gram-negative bacteria were the primary causative organisms, primarily due to their presence in the digestive tract. *Klebsiella pneumonia* 6 (27.3%), XDR *Acinetobacter baumannii* 3 (13.6%), and *E. coli* 3 (13.6%) were the primary causative organisms. In addition, Gram-positive organisms were present, including *MRSA* 4 (18.2%), Coagulase-negative *Staph* 2 (9.1%), and *Candida albicans* 4 (18.2%). Figure 1 shows the percentage of pathogens of DAI collectively.

## Discussion

A study conducted by the CDC regarding the efficacy of infection prevention and control measures in hospital settings found that almost 35% of HAI infections are preventable when using suitable approaches. Following this study, numerous researchers conducted investigations on the impact of different interventions aimed at reducing infection rates [6]. While the care bundle has been acknowledged as an effective method for reducing infection rates in many ICU settings, there is still a need for evidence of its application in LDLT settings.

The current study demonstrated that a care bundle could be successfully fulfilled in an LT unit, reducing more than 50% of device-associated infections (VAP, CAUTI, CLABSI) after the consistent implementation of all the bundle elements together, which aligns with the findings of Harbarth [13] and Umscheid [14].

Harbarth and his colleagues conducted a systematic review of the literature for studies using multiple interventions to provide a rough estimation of the number of preventable HAIs. They concluded that the reduction effect could range from 10 to 70%. This variation depends on the study design and setting, the infection rates, and the types at baseline [13]. Umscheid and his colleagues have recently demonstrated that it is achievable to prevent 65–70% of CLABSI and CAUTI cases and 55% of VAP cases. Among these, CLABSI is the most preventable and cost-effective [14].

In our study, CAUTI was the most common DAI. Consistent with our study, Parekh et al. found that CAUTI was the most prevailing cause of DAIs [15]. Conversely, Pouladfar et al.’s study found that VAPs were the most common DAIs (16.5% had VAP compared with 8.2% with CAUTI and 5.9% with CLABSI) [5].

In an extensive systematic review by Schreiber et al., the pooled incidence ratios associated with multimodal strategies to reduce HAI were 0.543 (95% CI, 0.435–0.662) for CAUTI, 0.459 (95% CI, 0.381–0.554) for CLABSI, and 0.553 (95% CI, 0.465–0.657) for VAP [16].

In contrast, Russell et al. found that the rate of CLABSI decreased from 4.1 to 1.6 in patients who underwent liver transplants between 2015 and 2016. The compliance rate for infection prevention measures was 95% [17]. Other studies by Blot et al. conducted additional research that yielded similar results. They implemented interventions to reduce CLABSI, such as preventive measures, process standardization, and bundle care awareness [18].

Most studies have reported XDR Gram-negative organisms as the leading cause of HAIs following LDLT. In a multicenter study on post-LDLT infections, Mukhtar and his colleagues found that *Pseudomonas aeruginosa* (26%) was the predominant bacterial species responsible for these infections. In 19% of the cases, *Klebsiella spp* was also identified, while *E. coli* was found in 16% of the cases. *Acinetobacter baumannii* was isolated in 8% of the cases, and MRSA was detected in 7.7% [19].

Another study by Montasser et al. found that the most common bacterial organisms isolated post-LDLT were gram-negative organisms with variation in the species identified. They reported *Acinetobacter baumannii* as the most common (19%), *Pseudomonas aeruginosa* (19%), followed by *Escherichia coli* in 11.1% [24]. The variations in the findings across studies could be attributed to differences in patients’ underlying diseases and types of HAIs [19–22].

Screening for bacterial colonization in patients undergoing LDLT allows for the identification of candidates for rapid and effective empirical antimicrobial treatment, limiting ineffective or broad-spectrum antibiotics and reducing antimicrobial resistance to potential pathogens [23].

Furthermore, prior studies performed by Yousef [25], Al-Faouri [26], and Galal et al. [27] enhanced the understanding of hand hygiene concept results in consistent adherence during the follow-up period.

The key advantages of bundle care approach included enhanced consistency through close monitoring and real-time feedback to immediately identify and resolve implementation gaps, increased compliance due to regular assessment of adherence to the bundle components, improved training that better equipped healthcare workers with the necessary knowledge and skills, and front-line ownership and engagement that fostered a culture of continuous learning and improvement. Collectively, these elements contributed to reducing the rate of DAI, ultimately leading to improved patient outcomes as demonstrated in single center and multicenter studies done by Gupta et al. and Rosenthal et al. [28, 29]

The key challenges of bundle care approach included ensuring consistent compliance, overcoming resistance to change, and sustaining improvements over time. The perceived complexity of the intervention, concerns about the design quality, and organizational factors such as structural characteristics, communication networks, and cultural norms that hindered implementation were the main barriers encountered in some studies [30]. Lack of readiness for change, tension for implementation, and the bundle incompatibility with existing workflows and stakeholder needs also posed significant barriers. Insufficient planning, lack of formally appointed implementation leaders, and limited engagement of champions further impeded the execution and evaluation of the implementation process [30].

In conclusion, This study demonstrates the effectiveness of utilizing the care bundle approach to reduce DAI in LDLT, especially in low socioeconomic countries with limited resources. By implementing a comprehensive set of evidence-based interventions, healthcare systems in these settings can effectively reduce the burden of DAI, enhance infection prevention strategies and improve patient outcomes in resource-constrained settings.

One of the study limitations was the small sample size, which can restrict the generalizability of the findings. Moreover, the infrequent occurrence of liver transplantation as a major surgical procedure posed challenges in obtaining a larger sample size, thereby limiting the ability to detect significant results. Collaborative initiatives and multi-center studies can be valuable effective strategies to achieve substantial number of cases. It is important to take these limitations into account when interpreting the findings of the study.

## Data Availability

Data is provided within the manuscript.
